# The CP312R protein of African swine fever virus inhibits host protein translation via the BiP/PERK/eIF2*α* pathway

**DOI:** 10.1186/s13567-025-01688-5

**Published:** 2026-02-25

**Authors:** Jingwen Dai, Pingping Zhou, Yanjin Wang, Haojie Ren, Kehui Zhang, Meilin Li, Hailiang Ge, Hongwei Cao, Jiaqi Li, Hao Deng, Wen-Rui He, Lian-Feng Li, Su Li, Hua-Ji Qiu

**Affiliations:** 1 State Key Laboratory of Animal Disease Control and Prevention, National High-Containment Facilities for Animal Disease Control and Prevention, National African Swine Fever Para-Reference Laboratory, Harbin Veterinary Research Institute, Chinese Academy of Agricultural Sciences, 678 Haping Road, Harbin, 150069 Heilongjiang China; 2 Harbin Medical University, Harbin, 150081 China; 3 International Joint Research Centre of National Animal Immunology, College of Veterinary Medicine, Henan Agricultural University, Zhengzhou, 450046 China

**Keywords:** African swine fever virus, CP312R protein, shutoff, PKR-like endoplasmic reticulum kinase, eukaryotic initiation factor 2*α*, heavy-chain-binding protein

## Abstract

African swine fever is a highly contagious, febrile, hemorrhagic, and often fatal disease of domestic pigs and wild boar, caused by African swine fever virus (ASFV). ASFV has evolved multiple strategies to suppress host antiviral responses, including the regulation of host translation shutoff for immune evasion. However, the intricate mechanisms of translation shutoff regulated by viral proteins remain largely unexplored. A previous study has shown that pCP312R—a structural protein of ASFV with 312 amino acids (aa)—inhibits host protein translation by binding to the ribosomal protein S27A (RPS27A). Here, we demonstrated that the RNA polymerase-like domain (aa 59–163) of pCP312R was essential for inhibiting protein synthesis. However, the RNA polymerase-like domain of pCP312R did not interact with RPS27A, implying the existence of a novel inhibitory mechanism. In this study, we showed that pCP312R specifically inhibited cellular protein synthesis at the stage of translation. Notably, overexpression of pCP312R reduced the expression of heavy-chain-binding protein (BiP). Furthermore, pCP312R induced the protein translation shutoff by promoting the phosphorylation of PKR-like endoplasmic reticulum kinase (PERK) and eukaryotic initiation factor 2*α* (eIF2*α*). Taken together, our study reveals that ASFV pCP312R inhibits cellular protein synthesis via the BiP/PERK/eIF2*α* pathway.

**Supplementary Information:**

The online version contains supplementary material available at 10.1186/s13567-025-01688-5.

## Introduction

African swine fever (ASF) is an acute, febrile, and fatal hemorrhagic disease caused by African swine fever virus (ASFV), which infects domestic pigs and wild boar with nearly 100% mortality in acute infection [1]. Currently, no safe and effective vaccines are commercially available globally except in Vietnam; the prevention and control of ASF therefore rely primarily on surveillance, stamping-out policies, and strict biosecurity measures. ASFV encodes over 160 proteins, which are primarily involved in viral genome replication, DNA damage repair, transcription, virion assembly, and immunoevasion [2, 3]. ASFV has evolved several strategies to hijack cellular proteins for the viral life cycle [4], and several viral proteins function in immunoevasion and competition of cellular resources [5–11].

It is well documented that the regulation of host cell translation by viral infections influences viral pathogenicity and immune evasion. The expression of many genes in eukaryotic cells is meticulously controlled at both the transcriptional and translational levels. Notably, translational regulation exerts a more rapid and direct influence on protein synthesis compared with transcriptional regulation. Specifically, most regulation events occur at the initiation stage of translation [12].

During the process of cellular protein synthesis, the eukaryotic initiation factor 2 (eIF2) plays a critical role by forming a ternary complex by binding to guanosine triphosphate (GTP) and initiator methionine-transfer RNA (Met-tRNAi). This ternary complex subsequently associates with the small ribosomal subunit. During translation initiation, upon recognition of the initiation codon “AUG”, the bound GTP is hydrolyzed and converted into guanosine diphosphate (GDP), leading to the release of eIF2 in its GDP-bound state that allows the assembly of the ribosome, which initiates the elongation phase of protein synthesis. eIF2 is composed of three subunits: *α*, *β*, and *γ*. Phosphorylation of eIF2*α* at serine 51 reduces the dissociation between eIF2 and its guanine nucleotide exchange factor eIF2B, thereby blocking the exchange between GTP and GDP. Enhancement of the binding of eIF2*α* to GDP can impede the function of the ternary complex, resulting in decreased global protein synthesis and increased transcription associated with stress [13].

Typically, viruses disrupt cellular protein synthesis through three distinct mechanisms: inhibition of eIF4A-mediated mRNA cap-binding, modulation of the mammalian target of rapamycin (mTOR) or the mitogen-activated protein kinase (MAPK) signaling pathway, and modulation of the phosphorylation of eIF2*α* and the activities of the eIF2–GTP–Met-tRNAi ternary complex [14]. The regulation of protein synthesis is typically accomplished through modulation of the phosphorylation of eIFs, such as eIF2*α* [15].

There exist four different kinases involved in the phosphorylation of eIF2*α*: protein kinase R (PKR), which can be activated by the double-stranded RNAs of viruses; PKR-like endoplasmic reticulum kinase (PERK), which is activated by endoplasmic reticulum stress (ERS); general control nonderepressible 2 (GCN2), which is activated by amino acid (aa) starvation; and the heme-regulated inhibitor, which is activated by heme deficiency [16, 17].

The endoplasmic reticulum (ER) is a distinct metabolic compartment and the primary organelle responsible for protein processing and modifications. Upon the accumulation of unfolded proteins within ER, the ER transmembrane proteins are released to trigger the unfolded protein response (UPR). These transmembrane proteins, with the N-terminus located in the ER and the C-terminus in the cytoplasm, serve as a bridge between the ER and the cytoplasm. Under physiological conditions, the N-termini of these ER transmembrane proteins are bound by heavy-chain-binding protein (BiP), which prevents protein aggregation. However, upon the occurrence of ERS, BiP is released to facilitate the aggregation of the transmembrane signaling proteins, thereby initiating the UPR [18–20].

The ASFV *CP312R* is an early-transcribed gene, which is located between positions 127303 and 128241 in the genome of the ASFV Pig/HLJ/18 (ASFV-WT) strain. It encodes the CP312R protein (pCP312R) of 312 aa [21]. The homology modeling analysis shows that pCP312R structurally resembles RNA polymerase. Furthermore, silencing *CP312R* resulted in reduced transcription of the *B646L* gene, which encodes p72, a major capsid protein of ASFV, suggesting that *CP312R* is critical to the ASFV life cycle. In the pRL-SV40 reporter system, pCP312R exerts robust inhibitory effects on cellular protein synthesis. In addition, the puromycin-labeling assay further confirmed that pCP312R overexpression reduces cellular protein synthesis. Overall, the data suggested that pCP312R functions as a potent inhibitor of cellular protein synthesis during ASFV infection.

In this study, we showed that ASFV infection significantly inhibits cellular protein synthesis, with pCP312R exhibiting a robust inhibitory effect on the process. Furthermore, we determined the precise stage at which pCP312R disrupts host protein synthesis and identified the pathways and targets involved.

## Materials and methods

### Cells, viruses, and antibodies

Human embryonic kidney 293T (HEK293T) cells and primary porcine alveolar macrophages (PAMs) were cultured in Dulbecco’s modified Eagle medium (DMEM; catalog no. C11995500BT, Gibco) or Roswell Park Memorial Institute (RPMI) 1640 medium (catalog no. C11875500BT, Gibco) supplemented with antibiotics–antimycotics (100 U/mL penicillin and 100 μg/mL streptomycin) (catalog no. 15140-122, Gibco) and 10% heat-inactivated fetal bovine sera (FBS; catalog no. 10099-141C, Gibco). The cells were maintained in a 37 °C incubator with 5% CO_2_. ASFV-WT was isolated from field samples from China as described previously [22]. ASFV-P61, a cell-adapted ASFV strain, replicates efficiently in HEK293T cells [23].

In-house swine anti-p54 polyclonal antibodies (PAbs) were as described previously [24]. Rabbit anti-pCP312R PAbs were produced by immunizing rabbits with the recombinant pCP312R produced in *Escherichia coli*. Mouse anti-p72 monoclonal antibody (MAb) (catalog no. Anti PPA 1BC11, INGENASA), mouse anti-puromycin MAb (catalog no. MABE343, Sigma-Aldrich), rabbit anti-*β*-actin PAbs (catalog no. AC026, ABclonal), mouse anti-Flag MAb (catalog no. M8823, Sigma-Aldrich), mouse anti-GST MAb (catalog no. K200006M, Solarbio), rabbit anti-Myc PAbs (catalog no. YN5506, ImmunoWay), rabbit anti-KDEL PAbs (catalog no. ab214714, Abcam), rabbit anti-BiP PAbs (catalog no. A0241, ABclonal), mouse anti-PERK MAb (catalog no. 3192S, CST), rabbit anti-p-PERK PAbs (catalog no. BS66100, Bioworld), rabbit anti-p-eIF2*α* PAbs (catalog no. 3398S, CST), and rabbit anti-eIF2*α* PAbs (catalog no. 9722S, CST) are commercially available. Lastly, 4′,6-diamidino-2-phenylindole (DAPI) (catalog no. C006, Solarbio) was purchased from Solarbio.

### Construction of plasmids and transfection of cells

The *CP312R* gene and its variants, including full-length *CP312R*, *CP312R-R* (encoding the RNA polymerase-like domain of pCP312R), and *CP312R-∆R* (encoding pCP312R lacking the RNA polymerase-like domain), were amplified via PCR from ASFV-WT genomic DNA using primers listed in Table 1. The genes were also cloned into pCAGGS-Flag to generate Flag-tagged constructs (pCA-CP312R-Flag, pCA-CP312R-R-Flag, and pCA-CP312R-∆R-Flag) and pGEX-6P-1 to generate GST-fused constructs (GST-CP312R, GST-CP312R-R, and GST-CP312R-∆R). In addition, the *RPS27A* gene was derived from the pCA-RPS27A-Flag plasmid [25] (kindly provided by Prof. Dongming Zhao) and cloned into pCAGGS-Myc to create pCA-RPS27A-Myc. Porcine *calnexin* was PCR-amplified and cloned into pDsRed2-C1 (Clontech) to generate pDsRed-calnexin.

**Table 1:** **The primers and siRNAs used in this study**

Primers/siRNAs	Sequences	Descriptions
Rluc-F	ATAACTGGTCCGCAGTGGTG	RT-qPCR for *Rluc*
Rluc-R	TAAGAAGAGGCCGCGTTACC	
hGAPDH-F	GAAGGTCGGAGTGAACGGATTT	RT-qPCR for human *GAPDH*
hGAPDH-R	TGGGTGGAATCATACTGGAACA	
pGAPDH-F	GAAGGTCGGAGTGAACGGATTT	RT-qPCR for pig *GAPDH*
pGAPDH-R	TGGGTGGAATCATACTGGAACA	
B646L-F	CTGCTCATGGTATCAATCTTATCGA	RT-qPCR for *B646L*
B646L-R	GATACCACAAGATC(AG)GCCGT	
CP204L-F	CGGTAGAATTGTTACGAC	RT-qPCR for *CP204L*
CP204L-R	TTCTTGAGCCTGATGTTC	
CP312R-F	TCCCAGTACCGATGAAGAGG	RT-qPCR for *CP312R*
CP312R-R	ATCTTCCAATGGCGGCGAGA	
DsRed-calnexin-F	GAATTCTATGGAAGGGAAGTGGTTGC	*calnexin* amplification for pDsRed2-C1
DsRed-calnexin-R	GGTACCTCACTCTCTTCGTGGCTTTC	
Flag-CP312R-F	CCGGAATTCGCCACCATGACTACACACATCTTTC	*CP312R* amplification for pCAGGS-Flag
Flag-CP312R-R	CCGCTCGAGAGCAATAGCAATCTGATT	
Flag-CP312R-R-F	ATCGATGCATGGTACCCGATGAAAGGCAAAAAAGCT	*CP312R-R* amplification for pCAGGS-Flag
Flag-CP312R-R-R	GTCTTTGTAGTCCTCGAGAGGACCTTTTTGCACAGC	
Flag-CP312R-∆R-1-F	ATCGATGCATGGTACCCGATGTTACTAGTAAAAATG	*CP312R-∆R* amplification for pCAGGS-Flag
Flag-CP312R-∆R-1-R	TGAGCCGCCGCCGCCTGAGCCGCCGCCGCCTACTATGACATTGACCGTAACATAT	
Flag-CP312R-∆R-2-F	GGCGGCGGCGGCTCAGGCGGCGGCGGCTCAGAAGCCATGAAAACGAAACATGTT	
Flag-CP312R-∆R-2-R	GTCTTTGTAGTCCTCGAGAGCAATAGCAATCTGATT	
siCP312R	GGAUUGAUGUGAACUCCAUdTdT	Sequences of siRNA against the *CP312R* gene
siNC	UUCUCCGAACGUGUCACGUTT	Sequences of non-targeting siRNA
GST-CP312R-F	CAGGGGCCCCTGGGATCCATGTTACTAGTAAAAATG	*CP312R* amplification for pGEX-6P-1
GST-CP312R-R	GAGTCGACCC GGGAATTCTT AAGCAATAGC AATCTG	
GST-CP312R-R-F	CCCCTGGGATCCCCGGAATTCATGAAAGGCAAAAAAGCTCCG	*CP312R-R* amplification for pGEX-6P-1
GST-CP312R-R-R	GTCACGATGCGGCCGCTCGAGAGGACCTTTTTGCACAGCGTC	
GST-CP312R-∆R-F	CCCCTGGGATCCCCGGAATTCATGTTACTAGTAAAAATGACT	*CP312R-∆R* amplification for pGEX-6P-1
GST-CP312R-∆R-R	GTCACGATGCGGCCGCTCGAGTTAAGCAATAGCAATCTGATT	

HEK293T cells were transfected with the indicated plasmids using the X-tremeGENE HP DNA transfection reagent (catalog no. 06366236001, Roche) in 6-well plates according to the manufacturer’s instructions. The cells were lysed with NP40 lysis buffer at 48 hours post-transfection (hpt).

### RNA extraction and RT-qPCR

Total RNA was extracted from the transfected HEK293T cells using Simply P total RNA extraction kit (catalog no. BSC52M1, BioFlux) and transcribed into cDNA using FastKing gDNA Dispelling RT SuperMix (catalog no. KR118-02, Tiangen). The transcription of the *Renilla* luciferase (*Rluc*) gene was quantified by reverse transcription-quantitative polymerase chain reaction (RT-qPCR) in a reaction mixture containing 10 μM primers targeting *Rluc* (Table 1) and 2 μL of the cDNA template under the conditions of 30 s at 95 °C, followed by 40 cycles of 95 °C for 15 s, 60 °C for 30 s, and 72 °C for 30 s. *GAPDH* was used as the internal control in this experiment.

### RNA interference assay

To determine the function of the *CP312R* gene in the life cycle of ASFV, PAMs in 24-well plates were transfected with 200 nM siRNA against the *CP312R* gene (siCP312R) using the X-tremeGENE siRNA transfection reagent (catalog no. 04476093001, Roche). After incubation for 6 h at 37 °C, the cells were infected with ASFV at a multiplicity of infection (MOI) of 0.1 for 18 h. Subsequently, the cells were subjected to RNA extraction by RNAiso Plus (catalog no. 9109, TaKaRa) according to the manufacturer’s instructions.

### Puromycin-labeling and western blotting analyses

PAMs were infected with ASFV-WT, while HEK293T cells were transfected with pCA-CP312R-Flag. The cells were pulsed with 3 μM puromycin for 30 min at the indicated h post-infection (hpi) or hpt and then subjected to western blotting analysis using mouse anti-puromycin MAb, swine anti-p54 PAbs, mouse anti-Flag MAb, mouse anti-GAPDH MAb, and rabbit anti-*β*-actin PAbs, respectively.

### GST pull-down assay

The GST-CP312R, GST-CP312R-R, GST-CP312R-∆R, or GST protein was incubated with the lysates from the cells transfected with the pCA-RPS27A-Myc plasmid at 4 °C for 12 h. The proteins pulled down by glutathione–sepharose 4B resin (catalog no. 71024800-EH, Cytiva) were analyzed by western blotting using anti-Myc PAbs or anti-GST MAb.

### Rluc reporter assay

HEK293T cells were cotransfected with pCA-CP312R-Flag and pRL-SV40 reporter plasmid. At 24 hpt, the cells were lysed by a Rluc assay lysis buffer (catalog no. E291A, Promega) for 30 min at 4 °C. After removal of cell debris by centrifugation at 4000 × *g* for 5 min, the Rluc activities were analyzed by the Rluc assay system (catalog no. E2810, Promega) according to the manufacturer’s protocols.

### Thapsigargin (Tg) treatment assay

HEK293T cells were transfected with an increasing amount of pCA-CP312R-Flag (0, 0.5, 1, 1.5, or 2 μg). At 12 hpt, the cells were treated with Tg (300 nM) for 8 h, and the phosphorylation of eIF2*α* was examined by western blotting analysis using the rabbit anti-p-eIF2*α* PAbs.

### Inhibitor treatment assays

HEK293T cells were transfected with an increasing amount of pCA-CP312R-Flag (0, 0.5, 0.8, or 1 μg). At 12 hpt, the cells were incubated with cycloheximide (CHX) at a final concentration of 100 μg/mL for 1 h, followed by transfection with pRL-SV40 (0.01 μg). At 12 or 24 hpt, the cells were collected for the examination of the mRNA transcription of *Rluc* by RT-qPCR.

HEK293T cells were transfected with pCA-CP312R-Flag (0, 1, 1.5, 2, or 2.5 μg). At 12 hpt, the cells were treated with ISRIB (0.5 μM) for another 12 h, and then the cells were collected for the expression of de novo protein synthesis by western blotting analysis.

HEK293T cells were transfected with pCAGGS or pCA-CP312R-Flag (1 μg each). At 18 hpt, the cells were incubated with 10 µM bafilomycin A1 (BafA1, an autolysosome inhibitor), 10 µM Z-VAD-FMK (ZVF, a caspase inhibitor), or 10 µM MG-132 (a proteasome inhibitor) for 6 h, and then the cells were collected and subjected to western blotting analysis.

### Statistical analysis

Statistical analyses were performed using GraphPad Prime 8.0. Differences between groups were analyzed via Student’s *t* test (*P < 0.05; **P < 0.01; ***P < 0.001; ns, not significant [*P* > 0.05]). Error bars denote the standard deviation of the mean from three independent experiments.

## Results

### ASFV pCP312R inhibits de novo protein synthesis

**Figure 1:**
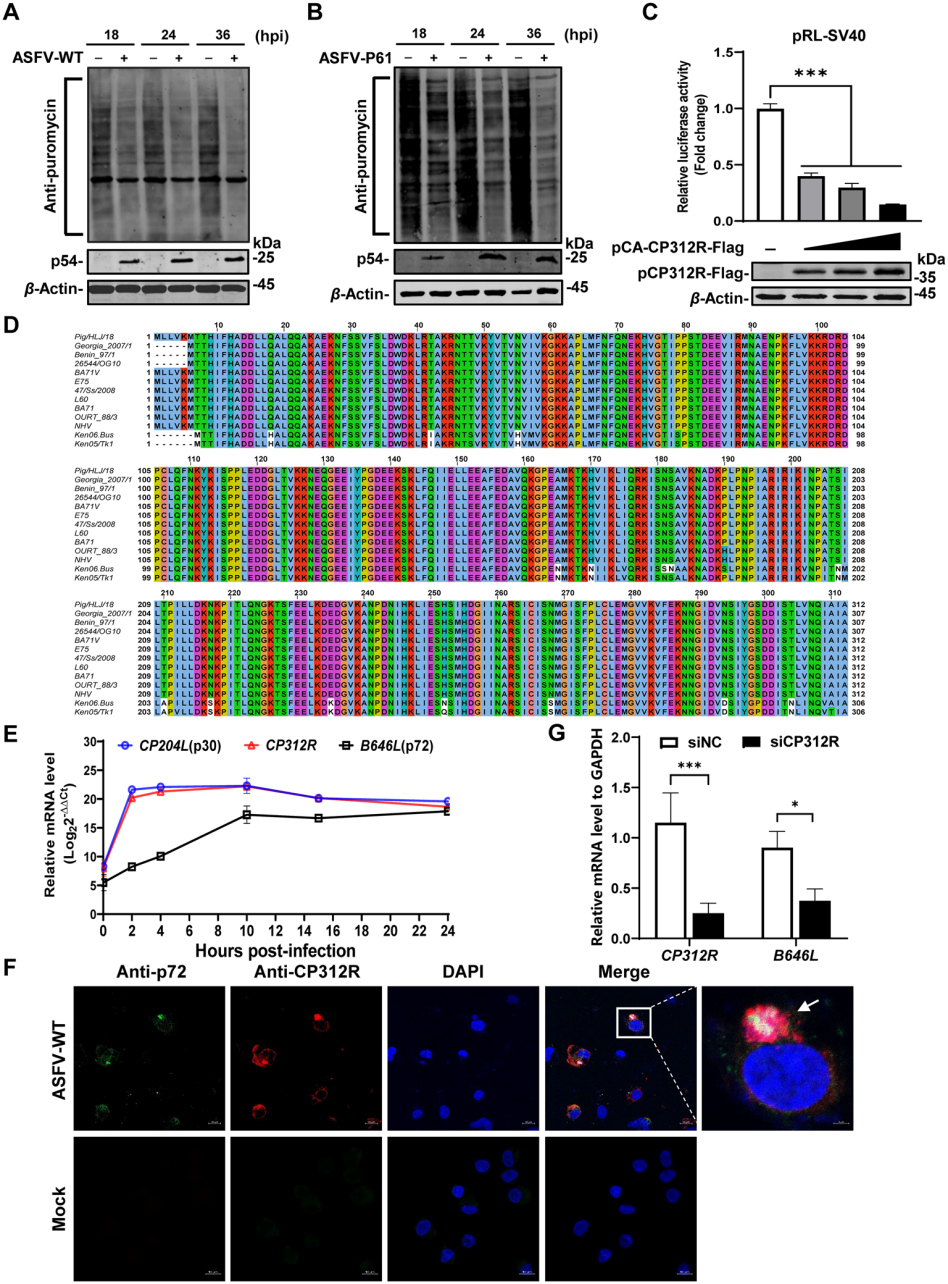
**ASFV pCP312R inhibits de novo** **protein** **synthesis. A** and **B** ASFV infection inhibits cellular protein synthesis in PAMs and HEK293T cells. PAMs infected with ASFV Pig/HLJ/18 (ASFV-WT) (MOI = 1) were pulsed with puromycin at 18, 24, or 36 h post-infection (hpi) and then subjected to western blotting analysis using anti-p54, anti-puromycin, or anti-*β*-actin antibodies (**A**). The inhibition effect was observed in ASFV-P61-infected HEK293T cells (**B**). **C** pCP312R inhibits the expression of *Renilla* luciferase (Rluc). HEK293T cells were cotransfected with pRL-SV40 (0.01 μg) and pCA-CP312R-Flag (0, 0.1, 0.2, or 0.5 μg). At 24 h post-transfection (hpt), the cells were examined by the Rluc assay system. **D** pCP312R is highly conserved among various ASFV isolates. Multiple sequence alignment analysis of pCP312R of 13 ASFV isolates was conducted on the Jalview software (v2.11.1.4) using the ClustalW algorithm. The 13 ASFV isolates are as follows: genotype I strains (Benin 97/1, E75, L60, BA71, OURT 88/3, and NHV), genotype II strains (Pig/HLJ/18, Georgia_2007/1, and 47/Ss/2008), genotype IX strain (Ken06.Bus), and genotype X strain (Ken05/Tk1). **E** The transcriptional dynamics of the *CP312R* gene. PAMs were infected with ASFV-WT (MOI = 5), and the average cycle threshold (Ct) values were examined at 2, 4, 10, 15, or 24 hpi by RT-qPCR using the primer pairs targeting the *CP312R*, *CP204L*, *B646L*, and *GAPDH* genes. **F** pCP312R is localized in the viral factories during ASFV infection. PAMs were infected with ASFV-WT (MOI = 1). At 18 hpi, the cells were immunostained with anti-pCP312R and anti-p72 antibodies. Cell nuclei were stained with DAPI. Scale bars: 2 μm. **G** The *CP312R* gene is required for the ASFV life cycle. PAMs were transfected with the siCP312R or siNC. At 6 hpt, the cells were infected with ASFV-WT (MOI = 0.1). At 18 hpi, the mRNA transcription of *CP312R* and *B646L* was examined by RT-qPCR.

Viruses have evolved various strategies to counteract cellular antiviral immunity, with inhibition of cellular protein synthesis being a significant tactic. To investigate the effects of ASFV infection on cellular protein synthesis, we used the puromycin-labeling assay to assess de novo protein synthesis in PAMs infected with ASFV-WT [22] and HEK293T cells infected with ASFV-P61 [23] at an MOI of 1 for 18, 24, or 36 h. The cells were incubated with 3 μM puromycin for 30 min. Subsequently, the cells were lysed with NP40 and subjected to western blotting analysis using an anti-puromycin MAb. The results showed that ASFV infection significantly inhibited the de novo synthesis of cellular proteins in a time-dependent manner in both PAMs (Figure 1A) and HEK293T cells (Figure 1B).

Given that ASFV inhibits the synthesis of cellular proteins, the pRL-SV40 reporter system, which harbors the *Rluc* gene under the control of the *SV40* promoter, was employed to identify the ASFV proteins responsible for the inhibition of protein synthesis (Additional file 1). We demonstrated that pCP312R significantly reduced *Rluc* expression in a dose-dependent manner (Figure 1C), implying that pCP312R inhibits protein expression.

To further characterize the role of pCP312R in ASFV replication, 13 ASFV isolates were examined by multiple sequence alignment using the Jalview software (v2.11.1.4). The data showed a very high degree of aa sequence similarity, with 88.14–99.68% homology among isolates containing the same or different forms of pCP312R (Figure 1D). To analyze the transcription kinetics of *CP312R*, total RNA was extracted from PAMs infected with ASFV-WT at an MOI of 5. The transcription of *CP312R* was quantified by RT-qPCR. The mRNA level of *CP312R* increased rapidly from 1 to 4 hpi, similar to that of *CP204L*, an early-transcribed gene encoding p30 (Figure 1E), indicating that *CP312R* is an early-transcribed gene of ASFV. To ascertain the subcellular localization of pCP312R during ASFV infection, PAMs were infected with ASFV-WT and examined using laser confocal microscopy. pCP312R was found to be localized within the viral factories (Figure 1F), as evidenced by the colocalization with p72, a capsid protein encoded by the ASFV *B646L* gene known to be localized in the viral factories [26]. The localization of pCP312R in the viral factories inspired us to explore the effects of pCP312R on viral replication. PAMs were transfected with the siCP312R and infected with ASFV at an MOI of 0.1. At 18 hpi, the cells were collected for the examination of viral transcripts using RT-qPCR. As shown in Figure 1G, the transcription of the *B646L* gene decreased upon *CP312R* knockdown, indicating that *CP312R* is required for the ASFV life cycle.

### pCP312R inhibits cellular protein synthesis by targeting the protein translation stage

**Figure 2:**
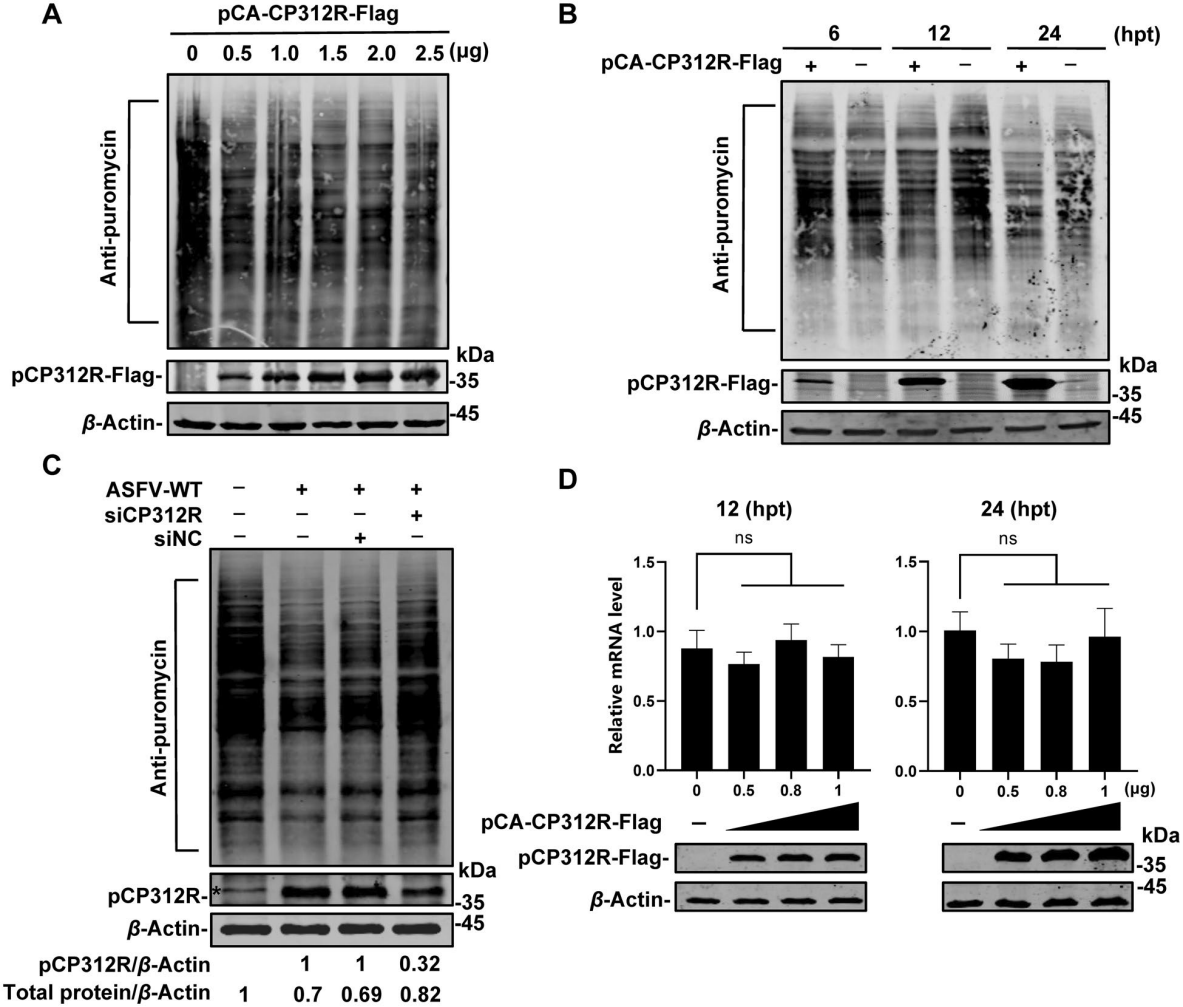
**The ASFV pCP312R inhibits cellular protein synthesis at the translation stage**. **A** and **B** pCP312R inhibits de novo protein synthesis. HEK293T cells were transfected with an increasing amount of pCA-CP312R-Flag (0, 0.5, 1, 1.5, 2, or 2.5 μg). At 18 h post-transfection (hpt), the cells were pulsed with 3 μM puromycin for 30 min and then subjected to western blotting analysis using anti-Flag, anti-puromycin, or anti-*β*-actin antibodies (**A**). HEK293T cells were transfected with pCAGGS or pCA-CP312R-Flag (1 μg each). The cells were pulsed with 3 μM puromycin for 30 min at 6, 12, or 24 hpt and then subjected to western blotting analysis using anti-Flag, anti-puromycin, or anti-*β*-actin antibodies (**B**). **C** Knockdown of *CP312R* partially restores cellular protein synthesis upon ASFV infection. PAMs were transfected with the siCP312R or a siNC. At 6 hpt, the cells were infected with ASFV-WT (MOI = 0.1). At 12 h post-infection, the cells were pulsed with 3 μM puromycin for 30 min and subjected to western blotting analysis using anti-CP312R, anti-puromycin, or anti-*β*-actin antibodies. *nonspecific protein band. **D** pCP312R does not alter mRNA transcription of the *Rluc* gene. The effects of the ectopically expressed pCP312R (0, 0.5, 0.8, or 1 μg) on the mRNA transcription of the *Rluc* gene were determined using RT-qPCR upon cycloheximide (CHX) treatment for 12 or 24 h. Error bars show the standard derivations (SDs) of the means from three independent experiments; ns, not significant.

To further verify the effects of pCP312R on cellular protein synthesis, HEK293T cells were transfected with pCA-CP312R-Flag, and the de novo synthesis of cellular proteins was determined by the puromycin-labeling assay. As shown in Figure 2A, the ectopically expressed pCP312R significantly inhibited the cellular protein synthesis in a dose-dependent manner. Moreover, the inhibition of host protein expression upon pCP312R overexpression was observed at different time points (Figure 2B), indicating that pCP312R inhibits cellular protein synthesis.

To further clarify the functional role of pCP312R in inhibiting cellular protein synthesis in the context of ASFV infection, siCP312R was used to knock down *CP312R* in the ASFV-infected PAMs. The results showed that the knockdown of *CP312R* partially restored cellular protein synthesis in the ASFV-infected PAMs (Figure 2C).

Protein expression is primarily categorized into two stages: transcription and translation [27]. To clarify which step(s) of protein synthesis is affected by pCP312R, the mRNA level of the *Rluc* gene in the presence of CHX was determined by RT-qPCR. The results showed that pCP312R did not affect *Rluc* mRNA synthesis at either 12 or 24 hpt (Figure 2D), indicating that pCP312R is involved in the translational stage rather than the mRNA transcriptional stage of protein synthesis.

### The RNA polymerase-like domain of pCP312R inhibits host protein synthesis

**Figure 3:**
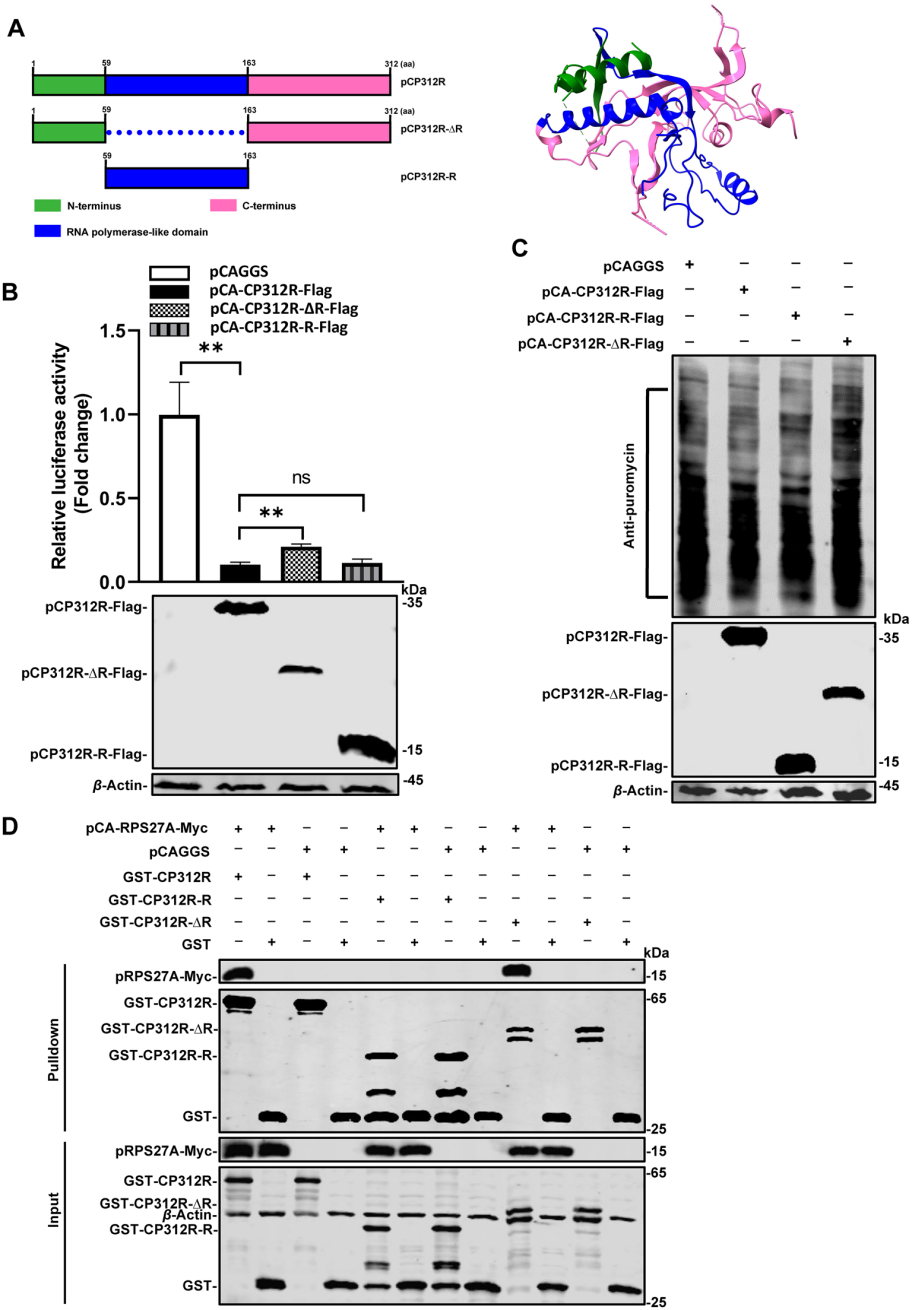
**The RNA polymerase-like domain of pCP312R is responsible for the inhibition of cellular protein synthesis**. **A** Schematic diagram of the pCP312R domains. The domains of pCP312R were analyzed by SWISS-MODEL [28], which revealed that the amino acids (aa) 59–163 on pCP312R display an RNA polymerase-like domain (left panel), and the three-dimensional structure diagram of pCP312R (based on PDB:7YEQ) was generated using the ChimeraX software [44] (right panel). The pCP312R-derived mutants, pCP312R-∆R (the mutant pCP312R lacking the RNA polymerase-like domain) and pCP312R-R (harboring the RNA polymerase-like domain of pCP312R), were each fused with a Flag tag in the C-terminus of the target genes. **B** and **C** The RNA polymerase-like domain of pCP312R exerts its inhibition of cellular protein synthesis. HEK293T cells were cotransfected with the pRL-SV40 plasmid (0.01 μg) harboring the *Rluc* gene controlled by the *SV40* promoter and each of the following plasmids: pCAGGS, pCA-CP312R-Flag, pCA-CP312R-R-Flag, or pCA-CP312R-∆R-Flag (0.5 μg each). At 24 h post-transfection (hpt), the cell lysates were subjected to Rluc assay (**B**). HEK293T cells were transfected with pCAGGS, pCA-CP312R-Flag, pCA-CP312R-R-Flag (harboring the RNA polymerase-like domain of pCP312R), or pCA-CP312R-∆R-Flag (the mutant pCP312R lacking the RNA polymerase-like domain) (1 μg each). The cells were pulsed with 3 μM puromycin for 30 min at 24 hpt and then subjected to western blotting analysis (**C**). Error bars show the SDs of the results from three independent experiments; ns, not significant. **D** The RNA polymerase-like domain of pCP312R does not interact with RPS27A. HEK293T cells were transfected with the pCA-RPS27A-Myc plasmid (1 μg). At 48 hpt, the cell lysates were subjected to GST pull-down assay, followed by western blotting analysis using anti-GST or anti-Myc antibodies.

To explore which domain on pCP312R is responsible for the inhibition of cellular protein synthesis, the domains of pCP312R were analyzed by SWISS-MODEL [28]. The data revealed that the domain spanning aa 59–163 of pCP312R was structurally similar to RNA polymerase (Figure 3A). Thus, the plasmid expressing the RNA polymerase-like domain of pCP312R (pCA-CP312R-R-Flag) and the plasmid expressing the mutant pCP312R lacking the RNA polymerase-like domain (pCA-CP312R-∆R-Flag) were constructed, respectively. By using the pRL-SV40 reporter system and the puromycin-labeling assay, we demonstrated that the RNA polymerase-like domain of pCP312R is responsible for the inhibition of protein synthesis. The inhibitory effects on Rluc activities and the protein synthesis of pCP312R-R were similar to those of pCP312R, whereas pCP312R-∆R did not display these inhibitory effects (Figures 3B, C). Collectively, the RNA polymerase-like domain of pCP312R is critical to its inhibition of cellular protein synthesis.

A recent study has demonstrated that pCP312R interacts with the host protein ribosomal protein S27A (RPS27A), thereby inhibiting host protein translation [25]. Therefore, we investigated the interaction between the RNA polymerase-like domain of pCP312R and RPS27A to determine whether the RNA polymerase-like domain, which is involved in translation shutoff in this study, can function independently of RPS27A. The results indicated that RPS27A did not interact with the RNA polymerase-like domain of pCP312R but instead interacted with the pCP312R mutant lacking this domain (Figure 3D). Consequently, the critical domain of pCP312R essential for inhibiting host protein synthesis functions independently of RPS27A, indicating the existence of a novel inhibitory mechanism that does not involve RPS27A.

### The ectopically expressed pCP312R reduces BiP expression

**Figure 4:**
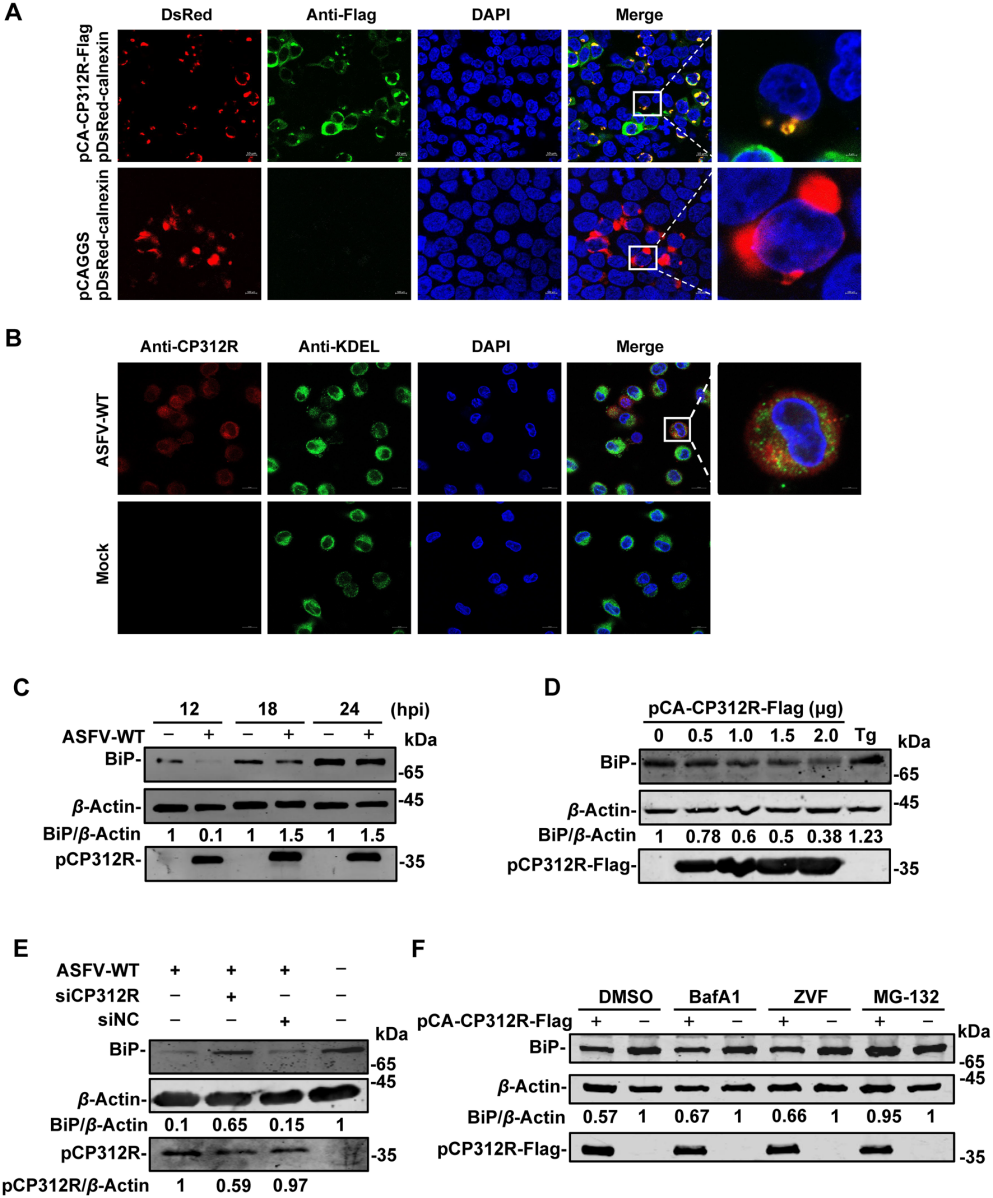
**The ectopically expressed pCP312R inhibits the expression of BiP**. **A** pCP312R is localized in the endoplasmic reticulum (ER) of the cell. HEK293T cells cotransfected with pCA-CP312R-Flag (0.2 μg) and pDsRed-calnexin (expressing calnexin, an ER marker) (0.5 μg). At 18 h post-transfection (hpt), the cells were analyzed by laser confocal microscopy. Scale bars: 2 μm. **B** pCP312R is localized in the ER in the ASFV-infected PAMs. PAMs were infected with ASFV-WT (MOI = 1). At 12 h post-infection (hpi), the cells were immunostained with anti-pCP312R and anti-KDEL antibodies. Cell nuclei were stained with DAPI. Scale bars: 2 μm. **C** ASFV infection induces in the differential expression of BiP. PAMs were infected with ASFV-WT (MOI = 1). At 12, 18, and 24 hpi, the cells were collected for western blotting analysis using anti-BiP, anti-pCP312R, or anti-*β*-actin antibodies. **D** The ectopic expression of pCP312R leads to a reduction in BiP expression. HEK293T cells were transfected with an increasing amount of pCA-CP312R-Flag (0, 0.5, 1, 1.5, or 2 μg). At 18 hpt, the cells were collected for western blotting analysis using anti-BiP, anti-Flag, or anti-*β*-actin antibodies. **E** Knockdown of *CP312R* partially restores the expression of BiP during ASFV infection. PAMs were transfected with the siCP312R or a siNC. At 6 hpt, the cells were infected with ASFV-WT (MOI = 0.1). At 12 hpi, the cells were collected for western blotting analysis using anti-CP312R, anti-BiP, or anti-*β*-actin antibodies. **F** pCP312R reduces BiP expression through the proteasomal pathway. HEK293T cells were transfected with pCAGGS or pCA-CP312R-Flag (1 μg each). At 18 hpt, the cells were incubated with 10 µM bafilomycin A1 (BafA1, an autolysosome inhibitor), 10 µM Z-VAD-FMK (ZVF, a caspase inhibitor), or 10 µM MG-132 (a proteasome inhibitor) for 6 h. The cells were subjected to western blotting analysis using anti-BiP, anti-Flag, or anti-*β*-actin antibodies.

To define the novel inhibitory mechanism, we investigated the organelle in which pCP312R exerts its inhibitory effects on host protein synthesis. HEK293T cells were cotransfected with pCA-CP312R-Flag and the plasmids expressing different organelle markers, including Lamp1 (the lysosome-associated membrane protein 1), Mito (a mitochondria marker), calnexin (an ER marker), or pEGFP-Golgi (the Golgi apparatus). The results showed that pCP312R was colocalized with calnexin, exhibiting a Pearson’s correlation coefficient of 0.75 (Figure 4A and Additional file 2). To examine the subcellular location of pCP312R, PAMs were infected with ASFV-WT, and the localization of pCP312R was examined by laser confocal scanning microscopy using rabbit anti-pCP312R or anti-KDEL (KDEL: the ER retention signal) PAbs. The results showed that pCP312R was localized in the ER (with a Pearson’s correlation coefficient of 0.74) (Figure 4B), indicating that pCP312R is localized in the ER in the context of ASFV infection.

Since BiP is a constitutively expressed resident protein of the ER in eukaryotic cells [29], we examined the expression of BiP in PAMs upon ASFV infection at various time points. The results revealed that ASFV infection can modulate the expression of BiP, with a rapid decline followed by a gradual increase (Figure 4C). Furthermore, the expression of pCP312R resulted in decreased BiP (Figure 4D), and ERS induced by ASFV infection was assessed by western blotting upon *CP312R* knockdown. The results demonstrated that knockdown of *CP312R* abolished the inhibition of BiP expression induced by ASFV infection (Figure 4E). To elucidate the mechanism by which pCP312R reduces BiP expression, HEK293T cells were transfected with pCA-CP312R-Flag, followed by treatment with BafA1 (an autolysosome inhibitor), ZVF (a caspase inhibitor), or MG-132 (a proteasome inhibitor). We found that the suppression of BiP expression by pCP312R was attenuated specifically upon treatment with the proteasome inhibitor MG-132 (Figure 4F), indicating that pCP312R reduces the expression of BiP through the proteasomal pathway. Furthermore, we transfected with different amounts of pCA-CP312R-Flag and demonstrated that MG-132 treatment consistently inhibited CP312R-mediated BiP degradation (Additional file 3).

### pCP312R induces host protein translation shutoff via the PERK/eIF2*α* pathway

**Figure 5:**
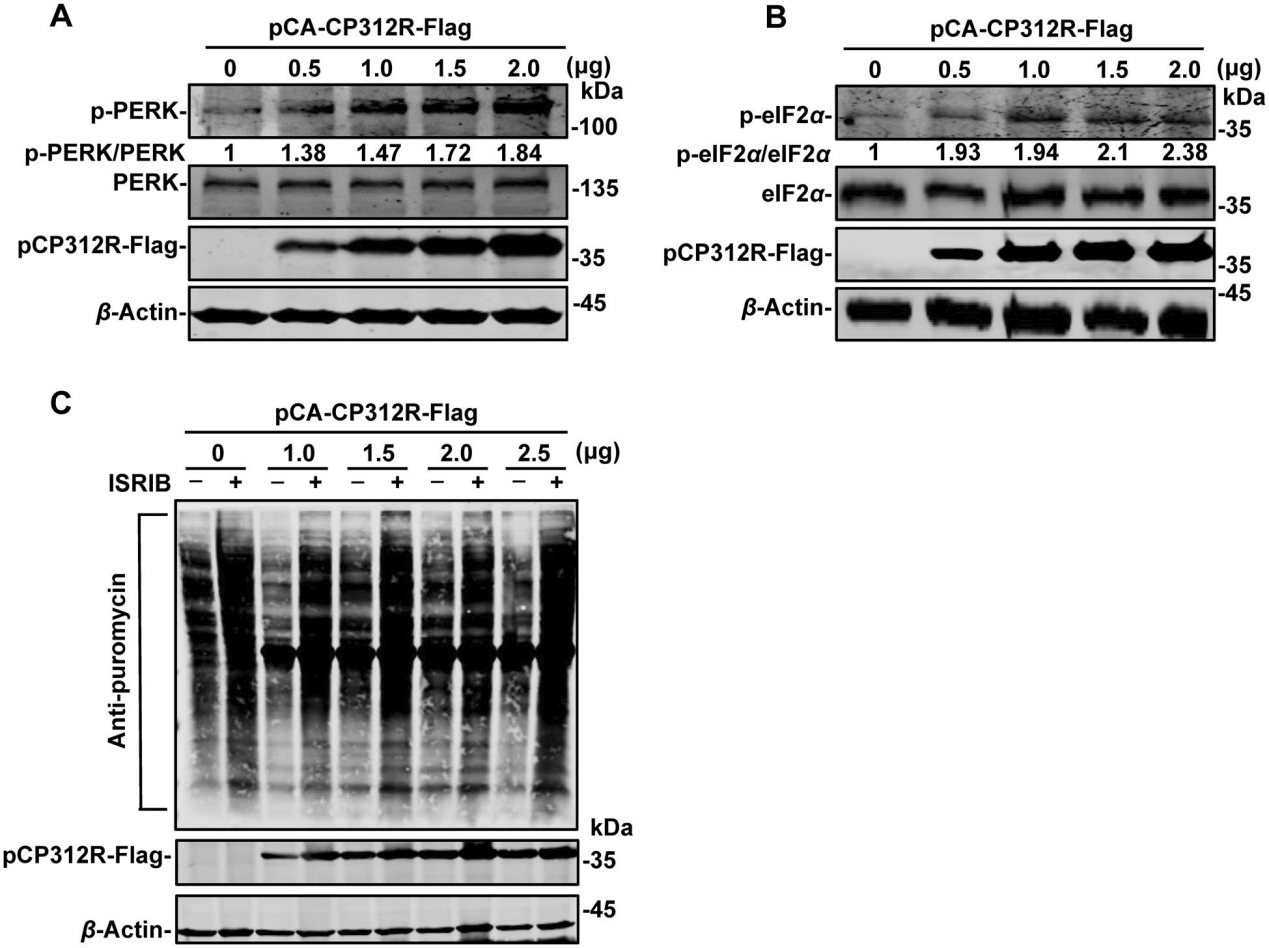
**pCP312R promotes the phosphorylation of PERK and eIF2*α***. **A** pCP312R promotes the phosphorylation of PERK in a dose-dependent manner. HEK293T cells were transfected with an increasing amount of pCA-CP312R-Flag (0, 0.5, 1, 1.5, or 2 μg). At 24 h post-transfection (hpt), the cells were collected for western blotting analysis using anti-Flag, anti-p-PERK (Thr982), anti-PERK, or anti-*β*-actin antibodies. **B** pCP312R promotes eIF2*α* phosphorylation in a dose-dependent manner. HEK293T cells were transfected with an increasing amount of pCA-CP312R-Flag (0, 0.5, 1, 1.5, or 2 μg). At 12 hpt, the cells were incubated with 300 nM thapsigargin (Tg) for 8 h, and then the cells were collected for western blotting analysis using anti-Flag, anti-p-eIF2*α*, anti-eIF2*α*, or anti-*β*-actin antibodies. **C** Inhibition of the cellular protein synthesis by pCP312R is attenuated by ISRIB. HEK293T cells were transfected with an increasing amount of pCA-CP312R-Flag (0, 1, 1.5, 2, or 2.5 μg). At 12 hpt, the cells were treated with ISRIB (0.5 μM) for another 12 h. The cells were pulsed with 3 μM puromycin for 30 min, followed by western blotting analysis using anti-Flag, anti-puromycin, or anti-*β*-actin antibodies.

As a major regulator of ERS, BiP binds to PERK under physiological conditions. In the presence of unfolded proteins, BiP dissociates from PERK, leading to the autophosphorylation of PERK and subsequent phosphorylation of eIF2*α* [30]. It has been shown that the phosphorylation of eIF2*α* is involved in the regulation of protein translation [8, 31]. To investigate the effects of pCP312R on the activation of PERK and the downstream eIF2*α*, we examined the phosphorylation of PERK and eIF2*α* upon pCP312R overexpression. The results indicated that pCP312R promoted the phosphorylation of PERK (Figure 5A) and eIF2*α* (Figure 5B) in a dose-dependent manner.

The integrated stress response (ISR) is activated in a variety of cellular states, rapidly reducing global protein synthesis while enhancing the translation of specific transcripts in response to the stress response. Various stress-sensitive kinases phosphorylate eIF2*α* at Ser51, mediating the ISR [32]. ISRIB (trans-isomer), a potent ISR inhibitor, can inhibit the phosphorylation of eIF2*α* [33], which can overrule part of the translational program imposed by PERK, relieving the general activation of translation [34]. Western blotting analysis revealed that ISRIB treatment significantly restored protein translation efficiency in pCP312R-expressing cells compared with mock-treated cells (Figure 5C). Taken together, these results suggest that pCP312R mediates protein translation shutoff via the PERK/eIF2*α* pathway.

## Discussion

ASFV has evolved multiple strategies to suppress host antiviral immunity, among which inhibiting cellular protein synthesis is critical [6, 8, 9, 35–41]. In this study, we demonstrated that pCP312R showed a high level of inhibition of cellular protein expression, as demonstrated by the pRL-SV40 reporter system and the puromycin-labeling assay.

Previously, Shen et al. conducted a comprehensive screening of the ASFV proteins involved in translation shutoff and identified viral proteins with robust inhibitory effects on host protein synthesis using the pRL-SV40 reporter system, including pMGF-360-9L, pMGF-360-10L, pMGF-360-11L, pA238L, pEP152R, pEP402R, pC122R, pC257L, pCP312R, pH171R, pQP509L, pE66L, pI243L, and pI329L [8]. By contrast, we identified the following viral proteins as exhibiting robust inhibitory effects (with a fold change of > 2) on host protein synthesis: pCP312R, pA224L, pB318L, pD250R, and pD205R (Additional file 1). Notably, pA224L and pB318L exhibited moderate inhibitory effects on host protein synthesis in Shen et al.’s study [8], whereas pCP312R displayed strong inhibitory effects in both studies. In addition, our results showed that pB117L, pDP96R, pH359L, and pMGF-110-3L exhibited weak inhibitory effects on host protein expression, whereas these proteins did not demonstrate such inhibitory capabilities in the previous study. Furthermore, pD129L, pC257L, pB263R, and pB407L exhibited similar trends in both studies, whereas pD205R did not. Overall, the present study demonstrated inhibitory effects consistent with the previous study. The minor differences in screening outcomes may be attributed to cellular states and experimental procedures. Importantly, the results of screening for host protein expression inhibition using the pRL-SV40 reporter system should be evaluated in combination with the puromycin-labeling assay.

**Figure 6:**
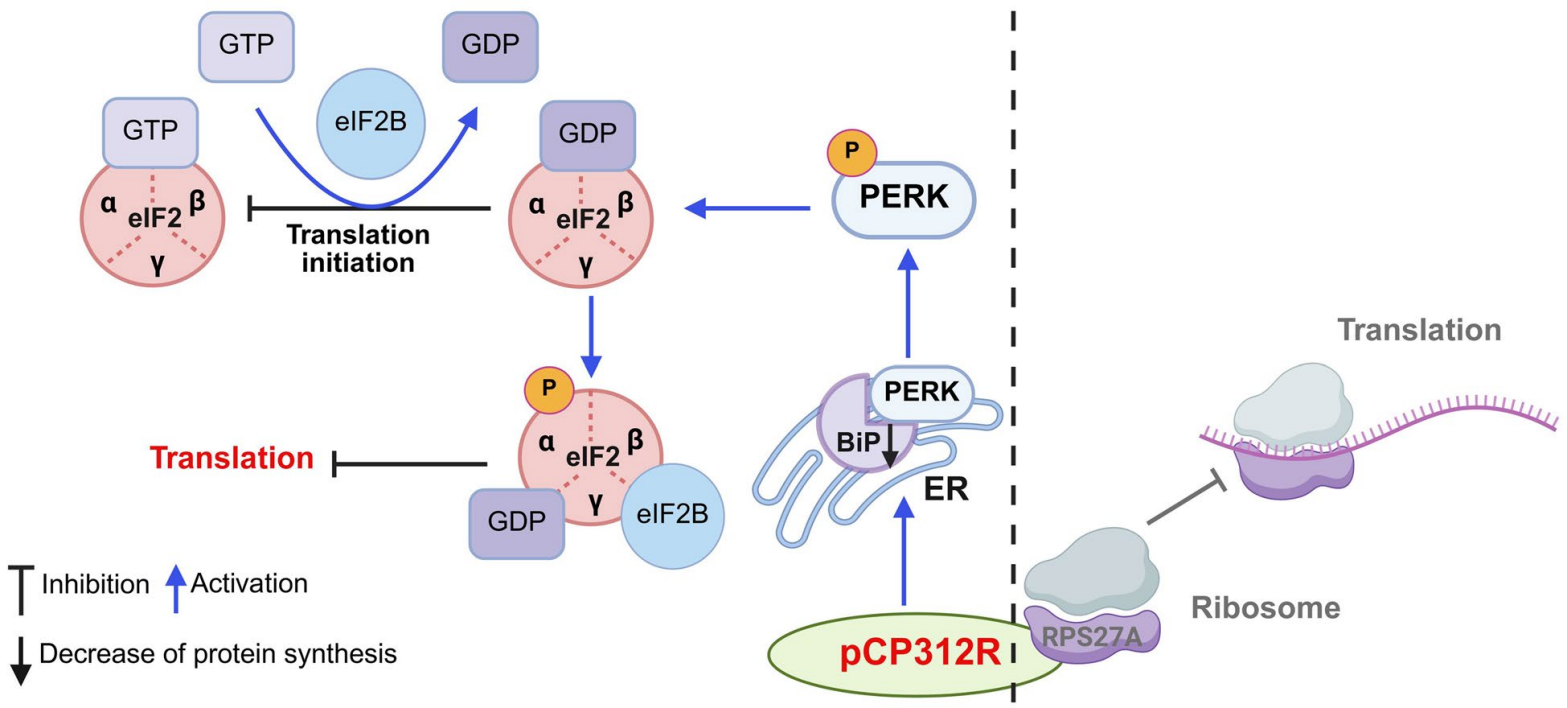
**A schematic model illustrating the inhibition of cellular protein synthesis by the ASFV pCP312R**. In addition to interaction with RPS27A [27], pCP312R downregulates the expression of heavy-chain-binding protein (BiP) and promotes the phosphorylation of the PKR-like endoplasmic reticulum kinase (PERK) and the eukaryotic initiation factor 2*α* (eIF2*α*), thereby inhibiting cellular protein synthesis.

pCP312R has been shown to inhibit cellular protein synthesis during the translation stage both in our group and other groups [25]. Generally, the eIF2 initiation complex integrates a variety of stress-related signals to regulate both global and specific mRNA translation in the intricate mechanism of cellular protein translation. eIF2 binds to GTP and Met-tRNAi to form a ternary complex, and then associates with the 40S ribosomal subunit, eIF1, eIF1A, eIF5, and eIF3 to form the 43S preinitiation complex (PIC) [42]. The 43S PIC scans the 5′ untranslated region (UTR) of mRNA for the AUG initiation codon. Upon recognizing the initiation codon AUG, eIF2 hydrolyzes GTP to GDP with the assistance of eIF5 and dissociates from mRNA, thereby facilitating the binding of 60S ribosomal subunit for elongation of the polypeptide chain. In this process, the phosphorylation of eIF2*α* is involved in the regulation of the translation pathway. Its phosphorylation leads to the dissociation of eIF2 from eIF2B, forming a dominant inactive complex that promotes the binding of eIF2*α* to GDP. The increased eIF2*α*–GDP limits the availability of the ternary complex, leading to a decrease in de novo protein synthesis (Figure 6).

In this study, we demonstrate that pCP312R is localized to the ER and reduces BiP synthesis, as shown in Figure 4B, D. ASFV is able to suppress the expression of BiP in the early stages of the virus life cycle, whereas the downregulated BiP expression induced by ASFV infection was gradually increased in the late stage of viral replication. Meanwhile, the ectopically expressed pCP312R can also inhibit the expression of BiP, which aligns with its role as an early protein in the ASFV life cycle.

Previously, the ASFV DP71L protein was shown to promote the dephosphorylation of eIF2*α* via the protein phosphatase 1 complex (PP1c), thereby reducing the expression of ATF4 and CHOP (downstream of eIF2*α*), and inhibit the UPR pathways owing to the ERS induced by ASFV infection [34]. Interestingly, we revealed that the expression of pCP312R promoted the phosphorylation of PERK and eIF2*α* in a dose-dependent manner (Figure 5A, B). Recently, the ASFV I215L protein (pI215L) has been shown to impair host protein stability. Specifically, pI215L functions at the post-translational modification level by regulating the expression of interferon regulatory factor 9 (IRF9) through the ubiquitin–proteasome system [43]. These two viral proteins collaboratively modulate host protein homeostasis by targeting protein synthesis and degradation. Furthermore, we have demonstrated that pCP312R downregulates the expression of BiP, thereby inducing ERS. In addition to suppressing global protein translation in host cells, pCP312R may also reduce BiP expression by promoting the degradation of preexisting BiP. This depletion of ER-resident BiP could subsequently induce ERS. This finding led us to investigate the pathway through which pCP312R promotes BiP degradation. Our findings revealed that pCP312R mediates BiP degradation via the proteasomal pathway.

During our manuscript preparation, pCP312R was reported to interact with host RPS27A to inhibit host protein translation [25]. In this study, we demonstrate that pCP312R indeed suppresses host protein synthesis through an alternative pathway, the BiP/PERK/eIF2*α* pathway, which functions independently of the pCP312R–RPS27A interaction. We further revealed that pCP312R is localized in the ER and engaged in the activation of the BiP/PERK/eIF2*α* pathway, indicating that pCP312R may regulate cellular protein synthesis through multiple distinct mechanisms.

Furthermore, we attempted to generate a *CP312R*-deficient ASFV mutant but the efforts were unsuccessful. Given that *CP312R* knockdown leads to reduced *B646L* expression, we proposed that the unsuccessful generation of the ASFV mutant with *CP312R* deletion might be related to impaired *B646L* functions. Next, we will try to generate an ASFV mutant with inducible expression of *CP312R* to clarify the exact functions of pCP312R.

In summary, we revealed that pCP312R induces host translation shutoff by the activation of the BiP/PERK/eIF2*α* pathway (Figure 6). This study not only elucidates the functional role of pCP312R in the interactions between ASFV and host cells but also provides a promising target for the development of antiviral strategies against ASF.

## Supplementary Information

**Additional file 1. Identification of the ASFV-encoded proteins that inhibit host protein synthesis.**HEK293T cells were cotransfected with pRL-SV40 (0.01 μg) encoding the *Renilla* luciferase (*Rluc*) gene controlled by the *SV40* promoter together with the ASFV protein-expressing plasmids (0.2 μg each). At 24 hpt, the cell lysates were examined for Rluc activities by the Rluc assay according to the manufacturer’s instructions.

**Additional file 2. pCP312R is not localized in lysosome, mitochondria, or Golgi of the cells.**HEK293T cells were cotransfected with pCA-CP312R-Flagand the plasmids expressing organelle markers, including pDsRed-Lamp1, pDsRed-Mito, or pEGFP-Golgi (0.5 μg each). At 24 hpt, the colocalization of pCP312R with the organelle markers in the cells was analyzed by laser confocal microscopy. Scale bars: 2 μm.

**Additional file 3. pCP312R reduces BiP expression through the proteasomal pathway. **HEK293T cells were transfected with pCA-CP312R-Flag of 0.5 (**A**) or 1.5 (**B**) µg. At 18 hpt, the cells were incubated with 10 µM bafilomycin A1, 10 µM Z-VAD-FMK, or 10 µM MG-132 for 6 h. The cells were collected and subjected to western blotting analysis using anti-BiP, anti-Flag, or anti-*β*-actin antibodies.

## Data Availability

All data and materials needed to evaluate the conclusions in the paper are present in the paper.
