# Supplementary figures and images for: The CP312R protein of African swine fever virus inhibits host protein translation via the BiP/PERK/eIF2α pathway
Source: Vet Res. 2026 Feb 25;57:46. doi: 10.1186/s13567-025-01688-5 (PMC13040927; doi:10.1186/s13567-025-01688-5)

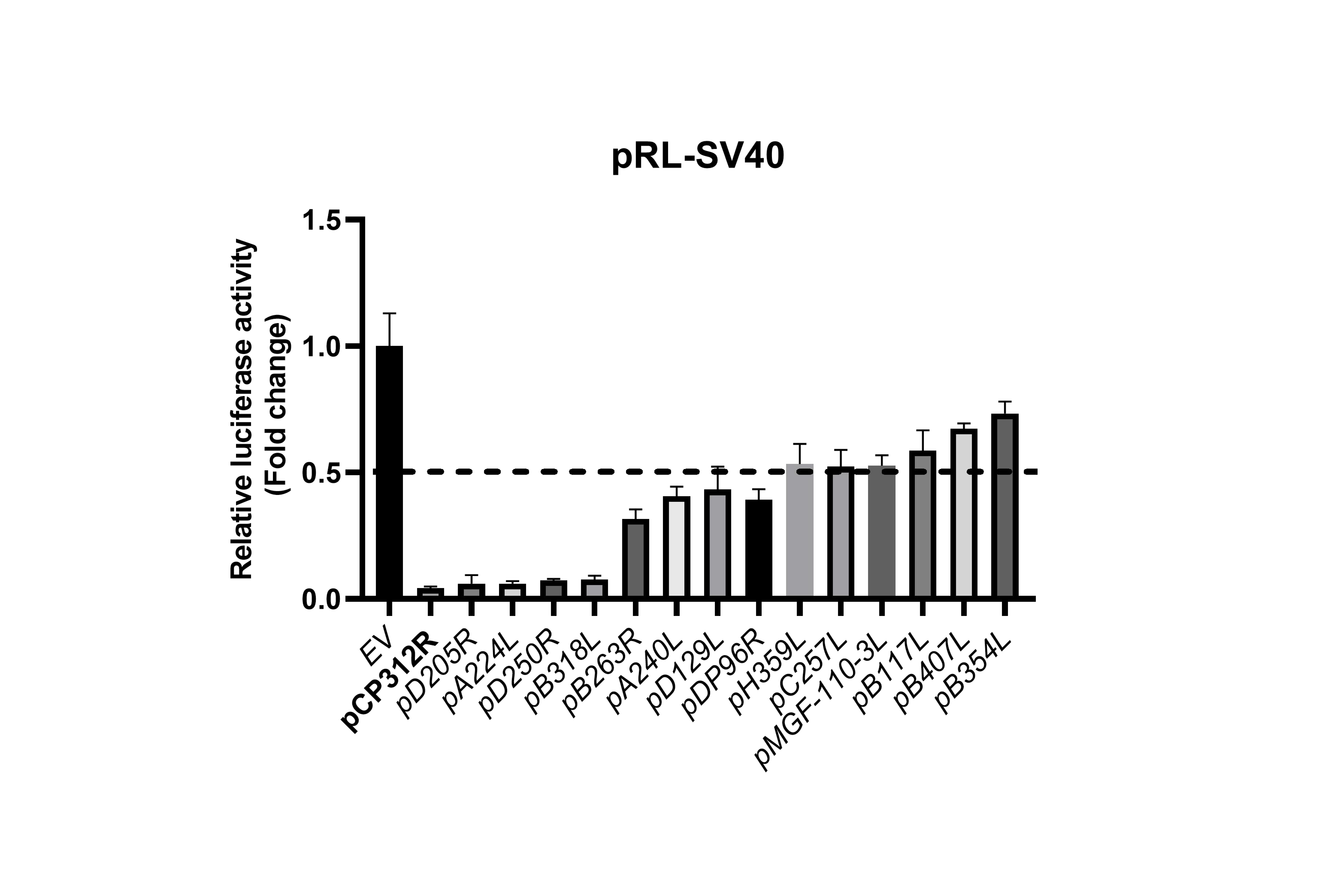

Supplement: Supplementary file 1 — Additional file 1. Identification of the ASFV-encoded proteins that inhibit host protein synthesis.HEK293T cells were cotransfected with pRL-SV40 (0.01 μg) encoding the Renilla luciferase (Rluc) gene controlled by the SV40 promoter together with the ASFV protein-expressing plasmids (0.2 μg each). At 24 hpt, the cell lysates were examined for Rluc activities by the Rluc assay according to the manufacturer’s instructions. [file 13567_2025_1688_MOESM1_ESM.tif]

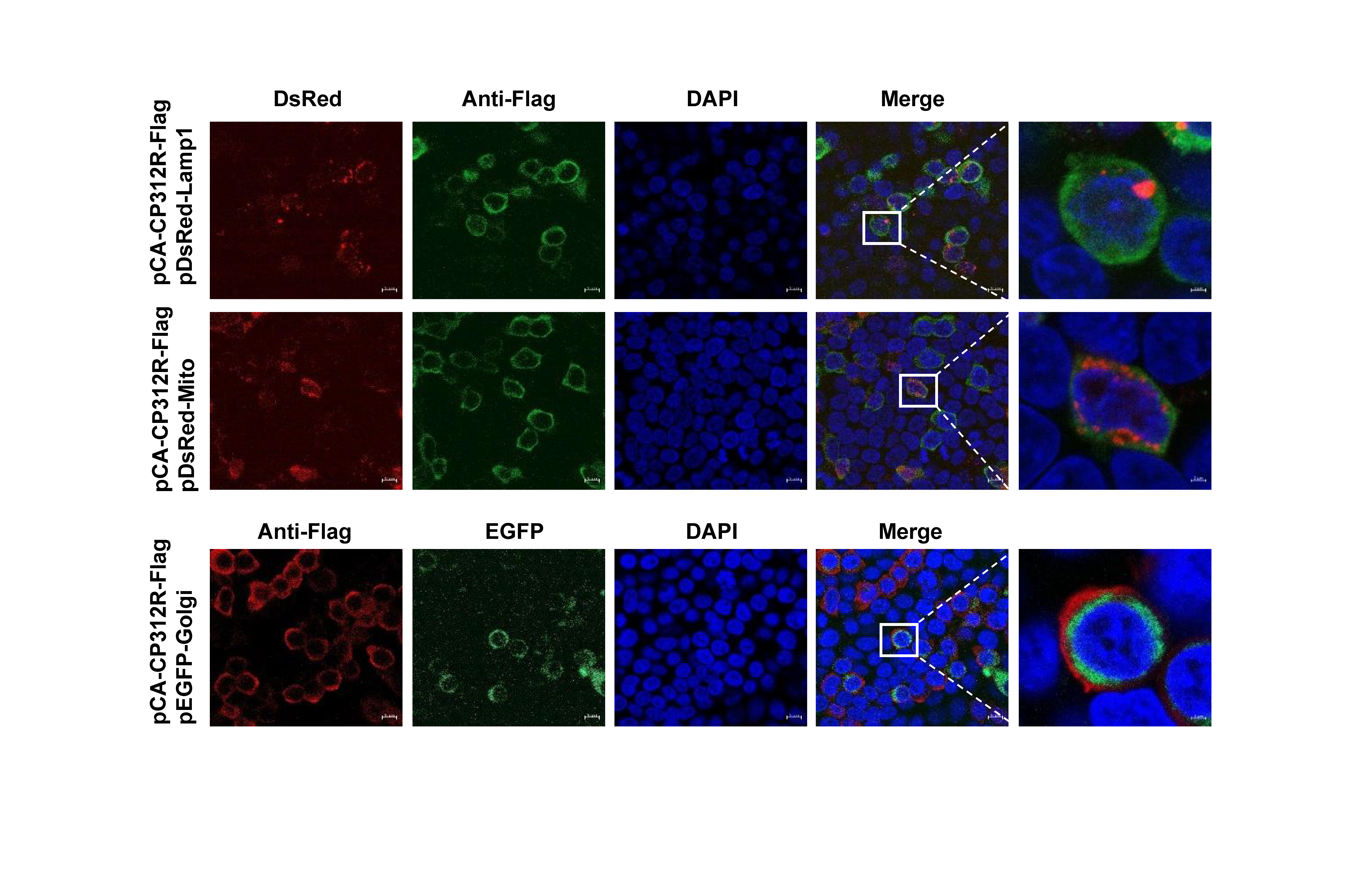

Supplement: Supplementary file 2 — Additional file 2. pCP312R is not localized in lysosome, mitochondria, or Golgi of the cells.HEK293T cells were cotransfected with pCA-CP312R-Flagand the plasmids expressing organelle markers, including pDsRed-Lamp1, pDsRed-Mito, or pEGFP-Golgi (0.5 μg each). At 24 hpt, the colocalization of pCP312R with the organelle markers in the cells was analyzed by laser confocal microscopy. Scale bars: 2 μm. [file 13567_2025_1688_MOESM2_ESM.tif]

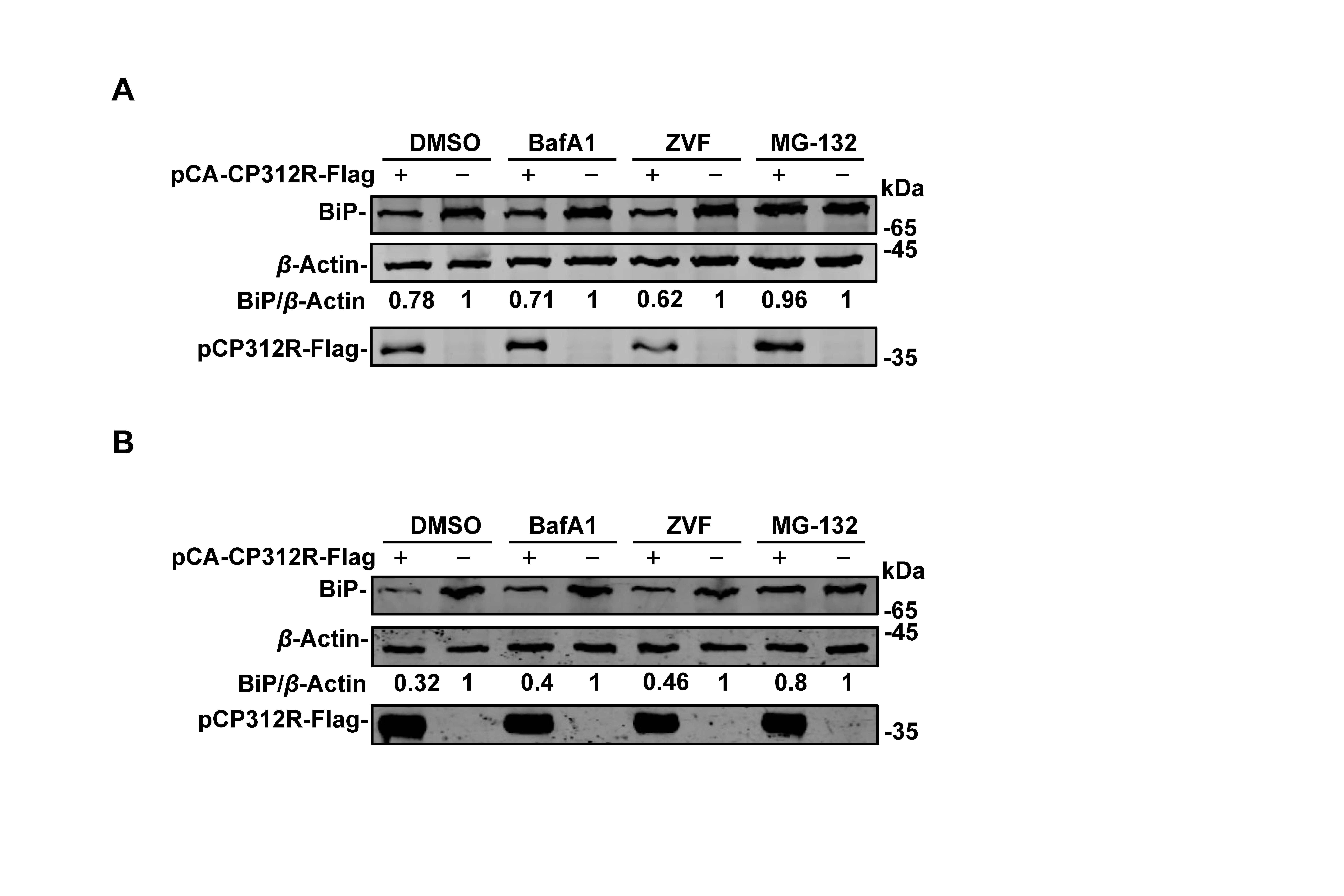

Supplement: Supplementary file 3 — Additional file 3. pCP312R reduces BiP expression through the proteasomal pathway. HEK293T cells were transfected with pCA-CP312R-Flag of 0.5 (A) or 1.5 (B) µg. At 18 hpt, the cells were incubated with 10 µM bafilomycin A1, 10 µM Z-VAD-FMK, or 10 µM MG-132 for 6 h. The cells were collected and subjected to western blotting analysis using anti-BiP, anti-Flag, or anti-β-actin antibodies. [file 13567_2025_1688_MOESM3_ESM.tif]
